# Functional Sex Reversal in Adult Gonochoristic Teleosts is Mediated by Ovarian Germline Stem Cell Plasticity and Somatic Cell Transdifferentiation

**DOI:** 10.1002/advs.77027

**Published:** 2026-08-15

**Authors:** Yang Yang, Liechao Yu, Wanchang Zhang, Gary Wessel, Ruiyi Chen, Weihua Hu, Dongdong Xu

**Affiliations:** ^1^ Zhejiang Marine Fisheries Research Institute Zhoushan China; ^2^ Zhejiang Ocean University Zhoushan China; ^3^ Zhoushan Field Comprehensive Scientific Observation and Research Station Ministry of Agriculture and Rural Affairs Zhoushan China; ^4^ School of Marine Sciences Ningbo University Ningbo China; ^5^ School of Life Sciences Nanchang University Nanchang China; ^6^ Department of Molecular and Cell Biology and Biochemistry Brown University Providence Rhode Island USA

**Keywords:** adult sex reversal, germline stem cells, gonochoristic vertebrates, sexual plasticity, somatic cell transdifferentiation, teleost fish

## Abstract

Sexual fate in vertebrates is generally considered developmentally fixed; however, teleost fishes exhibit remarkable flexibility in their reproductive systems. It remains unclear whether a fully differentiated gonochoristic vertebrate can undergo complete and functional sex reversal, or what molecular mechanisms could drive such reprogramming. Here, using the yellow drum (*Nibea albiflora*), a complete and functional female‐to‐male sex reversal is demonstrated in adulthood through three key mechanisms: (1) oocytes serve as guardians in the maintenance of ovarian fate, (2) ovarian germline stem cells retain spermatogenic potential and can produce sperm, and (3) ovarian somatic cells undergo complete transdifferentiation from granulosa/theca to Sertoli/Leydig lineages. This work provides the first evidence of functional female‐to‐male sex reversal in adult gonochoristic vertebrates, challenging existing paradigms about sexual fate fixation. Furthermore, these findings establish a novel experimental system for investigating vertebrate sexual plasticity and create a broadly representative model for sexual manipulation in teleost fishes.

## Graphical abstract

Oocytes play crucial roles in maintaining ovarian fate in adult gonochoristic fish. Targeted oocyte ablation with aromatase inhibition triggers the activation of surviving germline stem cells and the transdifferentiation of somatic cells from granoulosa/theca to Sertoli/Leydig lineages, inducing a complete and functional female‐to‐male sex reversal.

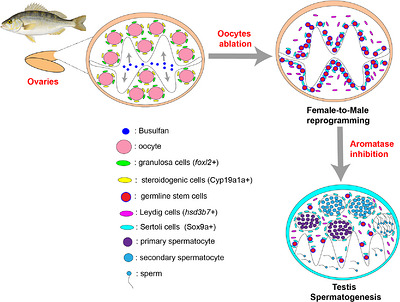

## 1 Introduction

Sexual fate in vertebrates has long been considered a stable, binary outcome, determined early in development and maintained for life [1]. In mammals, sex determination (SD) is initiated by a master regulator such as SRY, while mutually antagonistic transcriptional networks, centered on genes like FOXL2 in ovaries and DMRT1 in testes, ensure the lifelong maintenance of gonadal identity [2, 3]. However, recent genetic ablation studies in mice have revealed that adult gonads retain a latent plasticity [4, 5]. Although these interventions can trigger morphological sex reversal, they typically fail to restore full reproductive function, indicating that post‐developmental sexual identity remains under stringent control.

Teleost fishes, the most diverse group of vertebrates, exhibit the broadest diversity of SD systems, ranging from genotypic to environmental mechanisms, and including naturally occurring sequential hermaphroditism [6]. In hermaphroditic species, ovary‐to‐testis sex reversal follows a conserved developmental cascade involving oocyte degeneration, spermatogonial activation, and somatic remodeling [7, 8]. In contrast, gonochoristic teleost species with fixed sexes have traditionally been regarded as lacking such flexibility. However, recent studies using aromatase inhibition in model fishes such as zebrafish (*Danio rerio*) [9] and tilapia (*Oreochromis niloticus*) [10, 11] suggested that even fully differentiated adult gonads may retain unexpected plasticity. These findings raise a fundamental unresolved question: Can an adult gonochoristic vertebrate undergo complete female‐to‐male sex reversal? If so, what cellular relationships and regulatory hierarchies drive this transformation?

Sexual fate is governed by two fundamental components: germline identity and somatic lineage specialization. Across vertebrates, germ cells play key roles in directing gonadal sex differentiation. For instance, the depletion of early germ cells leads to the masculinization of chromosomally female medaka (*Oryzias latipes*) [12, 13] and zebrafish (*D. rerio*) [14, 15], whereas the sustained presence of oocytes reinforces ovarian fate. In parallel, gonadal somatic cells orchestrate both germline differentiation and the hormonal environment [16, 17]. Despite arising from bipotential origins, adult somatic lineages such as granulosa/theca and Sertoli/Leydig cells have been considered terminally committed in adulthood [18]. While studies in mammals demonstrate that disrupting genes like Foxl2 or Dmrt1 can reprogram somatic identity, such conversions typically fail to support full gametogenesis [4, 5], underscoring the challenge of reconstituting an intact and functional reproductive axis.

Here, we address these long‐standing questions using the yellow drum (*Nibea albiflora*), a marine gonochoristic teleost with a defined XX/XY chromosomal system and seasonal reproductive cycles [19, 20]. By integrating precise oocyte depletion, pharmacological manipulation of estrogen synthesis, molecular lineage tracking, and transcriptomic profiling, we reveal a previously unrecognized capacity for complete sexual fate reprogramming in an adult vertebrate. We demonstrate that oocytes serve as active guardians of ovarian identity: selective intra‐ovarian delivery of busulfan eliminates oocytes while preserving ovarian architecture, thereby destabilizing female fate and initiating a transcriptional shift toward testicular programs. Furthermore, we find that ovarian germline stem cell‐like (GSC‐like) cells persist following oocyte depletion and retain full spermatogenic potential, producing mature sperm under aromatase inhibition. Finally, we detect that ovarian somatic cells undergo extensive reprogramming, with granulosa and theca cells transdifferentiating into Sertoli and Leydig lineages, respectively. This organization recapitulates the cellular architecture of a testis and establishes complete spermatogenesis. Together, these results provide the first evidence of complete and fertile female‐to‐male sex reversal in an adult gonochoristic vertebrate. Our findings not only uncover unexpected sexual plasticity in adult teleosts, but also establish an experimentally tractable model for reproductive manipulation, with broad implications for germline biology, stem cell plasticity, and aquaculture biotechnology.

## 2 Results

### 2.1 Busulfan‐induced Oocyte Ablation in Ovaries of Female Yellow Drum

**FIGURE 1:**
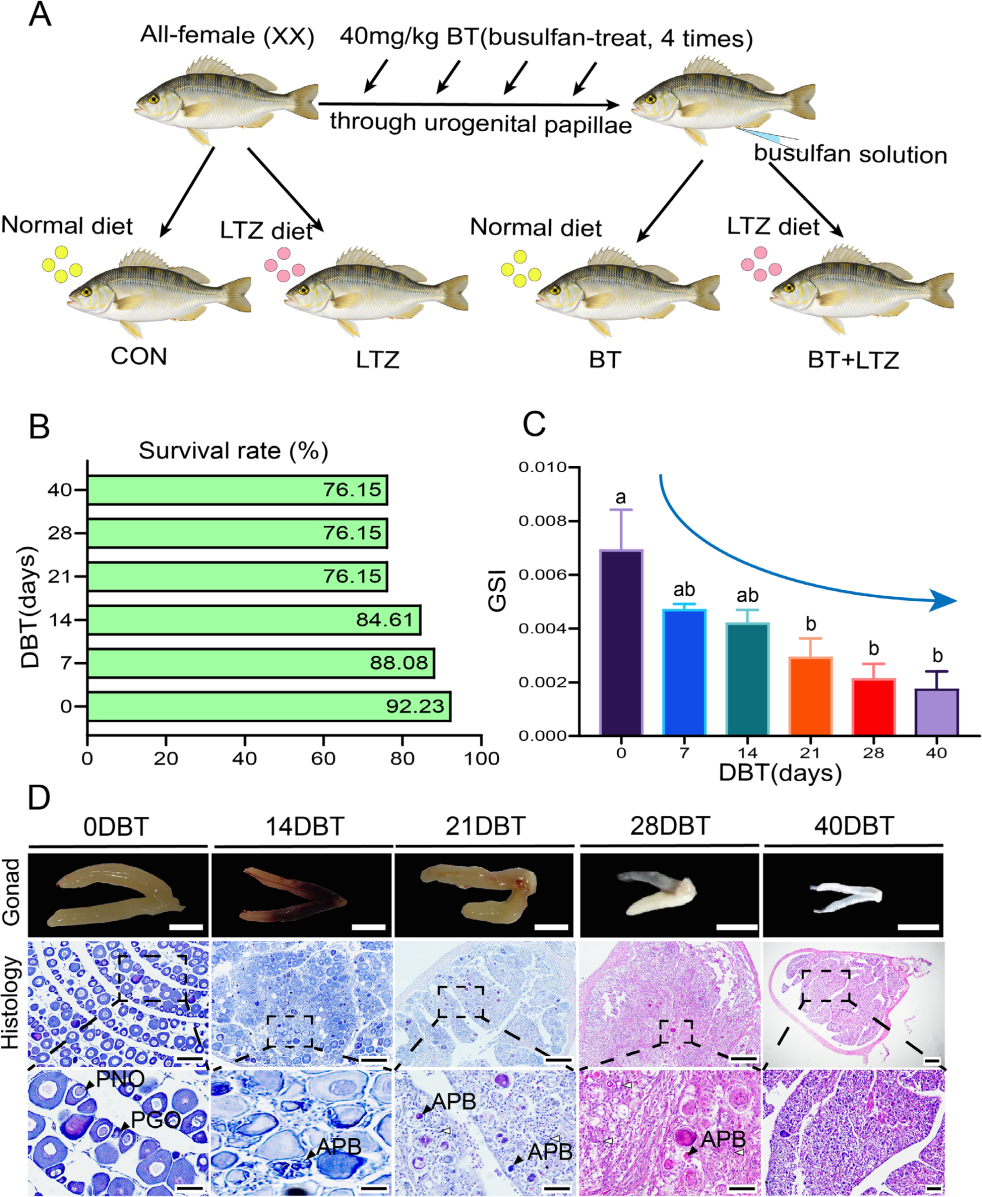
Busulfan‐induced oocyte ablation in ovaries of yellow drum (*Nibea albiflora*). A) Experimental design showing busulfan (BT) and letrozole (LTZ) treatments. All‐female (XX) yellow drum were treated with busulfan via intra‐ovarian injection, and injections were administered four times on days 0, 7, 14, and 21. Fish were divided into four groups: control (CON; genotypic fish fed a normal diet), letrozole‐treated (LTZ; genotypic females fed an LTZ diet), busulfan‐treated (BT; BT‐treated genotypic females fed a normal diet), and combined busulfan and letrozole‐treated (BT+LTZ, BT‐treated genotypic females fed an LTZ diet). B) Survival rates of fish at 0, 7, 14, 21, 28, and 40 days post‐busulfan treatment (DBT). A limited number of experimental fish died during the injection procedure as a result of operational stresses, including anesthesia and micropipette insertion; survival stabilized by 21 DBT. C) Gonadosomatic index (GSI) measurements of fish at 0, 7, 14, 21, 28, and 40 DBT. Data are presented as mean ± SEM. Different letters indicate significant differences (*p* < 0.05), n = 10 individuals from each sampling time. D) H&E staining of gonad sections shows oocyte ablation after busulfan treatment while maintaining ovarian architecture (ovigerous lamellae and ovarian cavity). Scale bars: Gonad, 1 cm; Histology, 300 and100 µm (high‐magnification insets). PNO, perinucleolar oocyte. PGO, primary growth oocyte. APB, apoptotic body.

In this study, busulfan solution (40 mg/kg BW) was injected with a glass micropipette into the ovaries via the ovarian duct, which opens into the urogenital papilla. To establish a sustained effect of the reagent, four injections were administered at 7‐day intervals (Figure 1A). Notably, busulfan treatment (BT) did not cause significant mortality in treated fish (Figure 1B). Both gonadosomatic index (GSI) and morphometric analysis demonstrated a pronounced reduction in gonadal mass post‐BT administration (Figure 1C,D). TUNEL staining indicated that oocyte ablation was mediated through apoptosis (Figure S1A,D). Histological examination revealed that degenerated oocytes and apoptotic bodies (APB) were first observed at the edges of ovigerous lamellae, and then massive somatic cells appeared (Figure 1D and Figure S1A). Finally, BT caused a complete depletion of oocytes, whereas ovarian structure was preserved, including intact ovigerous lamellae and an ovarian cavity (Figure 1D and Figure S1A). Furthermore, anti‐PCNA immunostaining highlighted sustained proliferative activity in somatic cells, which likely contributed to the maintenance of ovarian structural integrity after oocyte ablation (Figure S1B,E).

Immunostaining for Ddx4, a germ cell marker, showed that Ddx4‐positive signals were significantly reduced during BT (Figure S1C,F). However, some Ddx4‐positive GSC‐like cells remained detectable even following complete oocyte ablation. At 40 DBT, abundant GSC‐like cells were present in the edges of ovigerous lamellae (Figure S1C,F). These results indicate that while BT effectively eliminates differentiated oocytes, it spares the GSC‐like population, preserving their potential for germ cell regeneration.

### 2.2 LTZ‐induced Complete Secondary Sex Reversal in BT‐treated Ovaries

**FIGURE 2:**
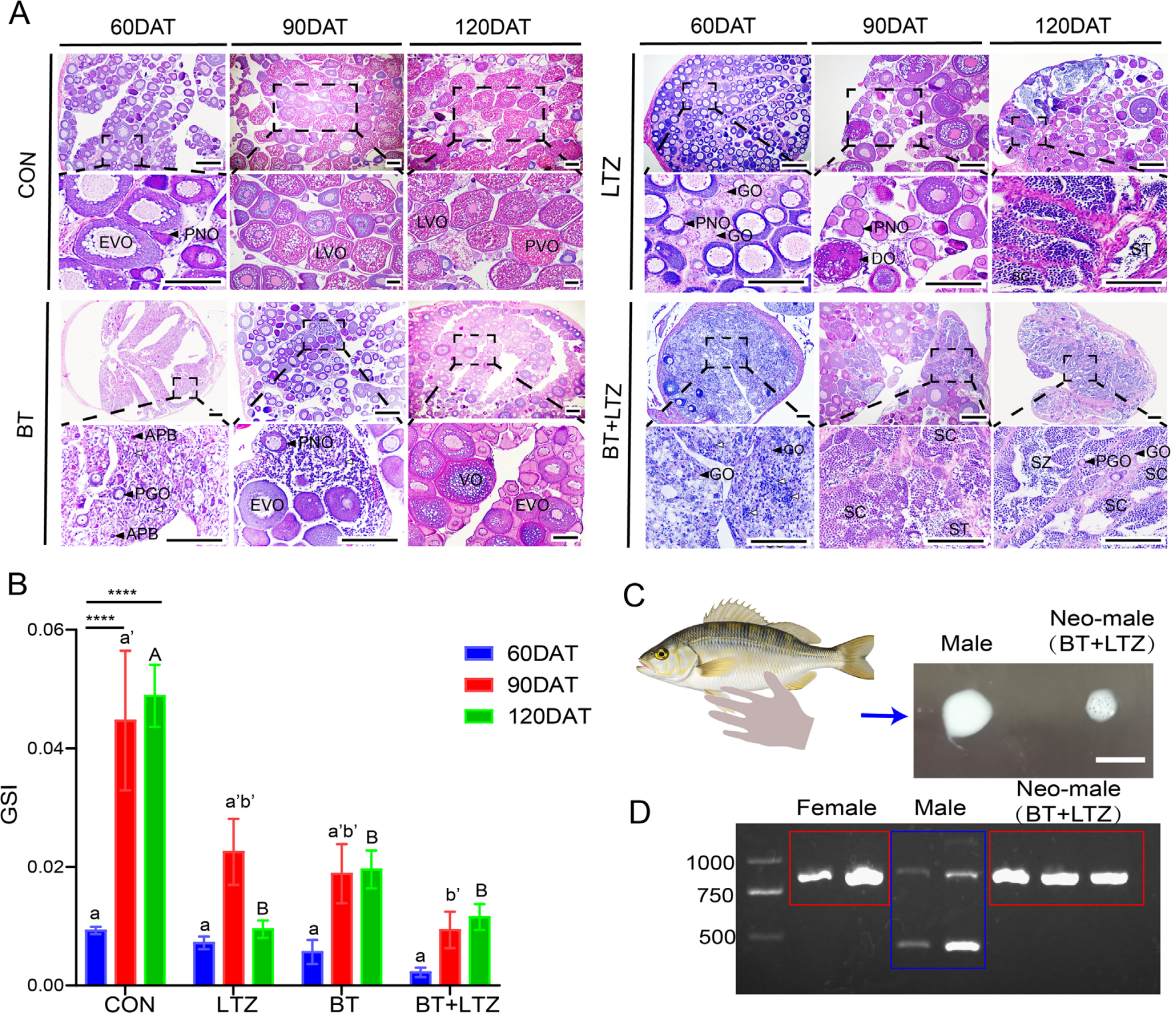
LTZ‐induced complete sex reversal in BT‐treated ovaries. A) H&E staining of gonad sections from control (CON), letrozole‐treated (LTZ), busulfan‐treated (BT), and combined BT+LTZ groups at 60, 90, and 120 days after treatment (DAT). The BT+LTZ group shows complete transformation into testicular structures. Scale bars: 200 and 50 µm (high‐magnification insets). EVO, early vitellogenetic oocyte. PNO, perinucleolar oocyte. LVO, late vitellogenetic oocyte. PVO, post‐vitellogenetic oocyte. GO, oogonia. DO, degenerated oocyte. PGO, primary growth oocyte. SC, spermatocytes. ST, spermatids. B) Gonadosomatic index (GSI) of fish for four experimental groups at 60, 90 and 120 DAT. Data are presented as mean ± SEM. Different letters represent significant differences. *p*< 0.05. *P*< 0.001(^***^) and *P*< 0.0001(^****^), n = 10 individuals each group from each sampling time. C) Functional sperm production in BT+LTZ fish. Semen collected at 180 DAT was translucent in sex‐reversed individuals compared with whitish semen from normal males. Scale bar: 1 cm. D) Genotyping confirmed that sex‐reversed individuals were genetic females. A single electrophoretic band (879 bp) for genotype‐females (XX) and two bands (879 and 449 bp) for genotype‐males (XY).

Letrozole (LTZ), a non‐steroidal aromatase inhibitor containing a triazole functional group, is widely employed to induce masculinization in teleost fish during early undifferentiated gonadal stages [21, 22]. To investigate whether LTZ induces secondary sex reversal in BT‐treated females, we conducted histological examinations to investigate gonadal fate. BT+LTZ‐treated gonads exhibited fully developed testicular structures with spermatocytes and spermatids, whereas control LTZ‐treated gonads showed only scattered spermatocytes and spermatids along the periphery of ovigerous lamellae (Figure 2A). These observations indicate that LTZ successfully induced complete sex reversal in combination with BT‐treated fish. In contrast, BT‐only treated ovaries displayed progressive oocyte reappearance at 90–120 days after treatment (DAT), indicating resumed oogenesis (Figure 2A). The analysis of GSI values confirmed significant suppression of gonadal development in both LTZ‐ and BT‐treated groups compared to controls (Figure 2B).

To assess the functionality of the sex‐reversed gonads, semen (milt) was collected from normal males and sex‐reversed individuals in the LTZ+BT at 180 DAT by gentle abdominal pressure. As shown in Figure 2C, milt from normal males appeared whitish, while milt from sex‐reversed males was translucent. Genotypic analysis using sex‐specific molecular markers confirmed that the sex‐reversed male individuals were genotypic females (neo‐males). Additional investigations through TUNEL staining and anti‐PCNA immunostaining demonstrated active germ cell proliferation coupled with somatic cell apoptosis during the reproductive phase, suggesting continuous gonadal remodeling in LTZ‐induced sex reversal (Figure S2).

### 2.3 Gene Expression Signatures Reveal Ovary‐to‐testis Transdifferentiation

**FIGURE 3:**
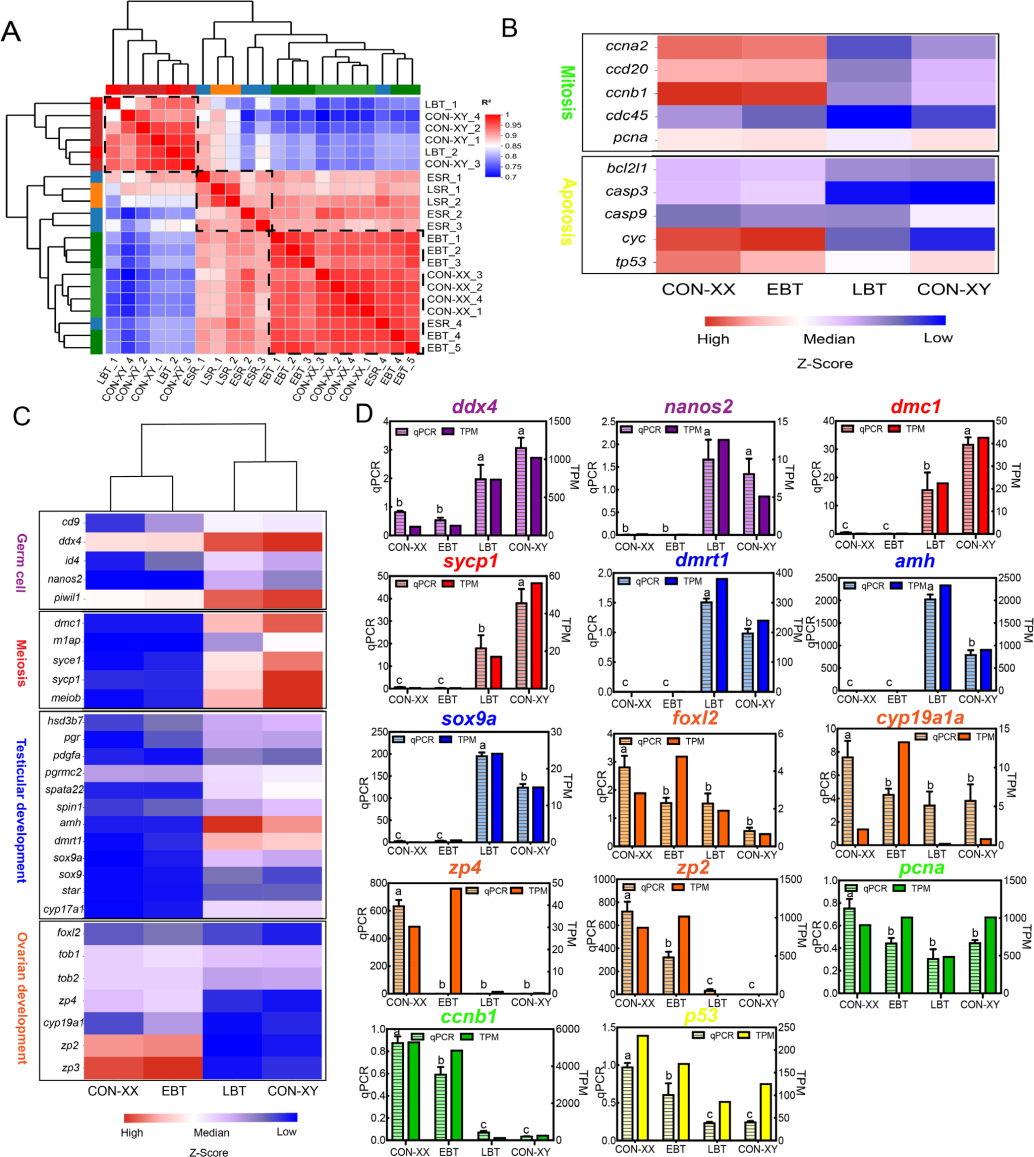
Transcriptomic analysis reveals gonadal masculinization after oocyte depletion. A) Hierarchical clustering and correlation analysis of 21 RNA‐seq samples from control female (CON‐XX), early busulfan‐treated (EBT, 21 and 28 DBT), late busulfan‐treated (LBT, 40 DBT), early sex reversal (ESR), late sex reversal (LSR), and control male (CON‐XY) gonads. B) Heatmap displaying genes enriched in mitosis and apoptosis. C) Expression levels of specific mRNAs related to germ cell, meiosis, testicular development, and ovarian development. D) RT‐qPCR validation and RNA‐seq expression values (TPM) for selected genes in control XX and XY gonads, EBT, and LBT gonads. Data are presented as mean ± SEM. Different letters indicate significant differences (*p*< 0.05), n = 3 each from three independent samples.

To elucidate the mechanism underlying ovary‐to‐testis transdifferentiation, we analyzed gene expression profiling (DE‐mRNAseq) across six groups: control ovaries (CON‐XX), control testes (CON‐XY), early‐stage BT‐treated ovaries (EBT), late‐stage BT‐treated ovaries (LBT), early‐stage BT+LTZ‐treated gonads (ESR), and late‐stage BT+LTZ‐treated gonads (LSR) (Figure 3 and Figure S3). Principal components analysis (PCA) and hierarchical clustering and correlation analysis were employed to visualize global expression patterns (Figure 3A and Figure S3A). The samples exhibited group‐specific clustering: CON‐XX and EBT samples clustered closely together, ESR and LSR samples formed a distinct intermediated cluster, and LBT samples clustered tightly with CON‐XY testes.

To characterize group‐specific transcriptional signatures during BT, the top 500 transcripts from EBT, LBT, CON‐XX and CON‐XY groups were subjected to Venn and Gene Ontology (GO) functional enrichment analyses (Figure S3B,C). Venn analysis revealed a ∼50% overlap in genes between the CON‐XX and EBT groups, and between the LBT and CON‐XY groups, respectively. GO terms analysis also demonstrated highly concordant functional terms between CON‐XX and EBT, and correspondingly between LBT and CON‐XY. This suggested that early‐stage oocyte‐depleted gonads largely retain a differentiated ovarian transcriptomic identity, whereas advanced oocyte depletion drives a transition toward a testicular expression program. Differential expression gene (DEG) analysis identified 2416 upregulated and 262 downregulated genes in the EBT compared to CON‐XX (Figure S4A). In contrast, the transition from EBT to LBT was characterized by profound transcriptomic remodeling, with 4356 upregulated and 3903 downregulated genes (Figure S4A). DEGs elevated in the EBT group (relative to CON‐XX) were associated with the immune system process, regulation of immune system process, and immune receptor activity (Figure S4B). Consistent with the correlation analysis, EBT ovaries retained ovarian transcriptional identity, with high expression of ovarian development‐related genes (*cyp19a1*, *foxl2*, *zp2* and *zp3)* (Figure 3C,D). These same gonads also exhibited elevated expression of key apoptosis regulators (*typ53*, *bcl2l1*, *casp3*, *casp9* and *cyc)* and mitosis genes (*ccna2*, *ccd20*, *ccnb1*, *cdc45* and *pcna*) (Figure 3B,D), reflecting a dynamic balance of cell death and proliferation during early gonadal remodeling. Notably, an upregulated expression of germline stem cell markers (*cd9* and *id4*) was initially observed in EBT. Although the meiotic germ cell markers (*dmc1* and *sycp1*) became nearly undetectable in the EBT group, weak expression of *ddx4* persisted. This indicates that while the vast majority of germ cells were ablated, a resident population of GSC‐like cells likely survived.

Conversely, DEGs upregulated in LBT relative to EBT were enriched for male‐biased reproductive functions, particularly reproductive process and meiotic cell cycle process (Figure S4D). Expression profiling of specific gene clusters revealed that transcripts significantly downregulated during the EBT‐to‐LBT transition (subcluster‐1, 3147 genes) were highly expressed in both CON‐XX and EBT groups. In parallel, genes upregulated during this transition (subcluster‐1, 4342 genes) exhibited high expression exclusively in LBT and CON‐XY gonads (Figure S4E,F). Within the “reproductive process” category, transcript patterns confirmed that *cd9*, *wbp2*, *pgr* and *si:ch211‐225h24.2* were among the earliest transcripts altered following initial oocyte depletion in the EBT (Figure S4G). In contrast, LBT gonads displayed testis‐like transcriptional reprogramming, marked by germline stem cell markers (*cd9*, *ddx4*, *id4*, *nanos2* and *piwil4*), meiosis‐related genes (*dmc1*, *m1ap*, *syce1*, *sycp1* and *meiob*) and testicular development‐related genes (*hsd3b7*, *amh*, *dmrt1*, *sox9a* and *cyp17a1*) (Figure 3C,D and Figure S4G). This sequential shift from ovarian to testicular gene expression profiles demonstrates BT‐induced gonadal masculinization through oocyte depletion‐mediated transcriptional remodeling.

**FIGURE 4:**
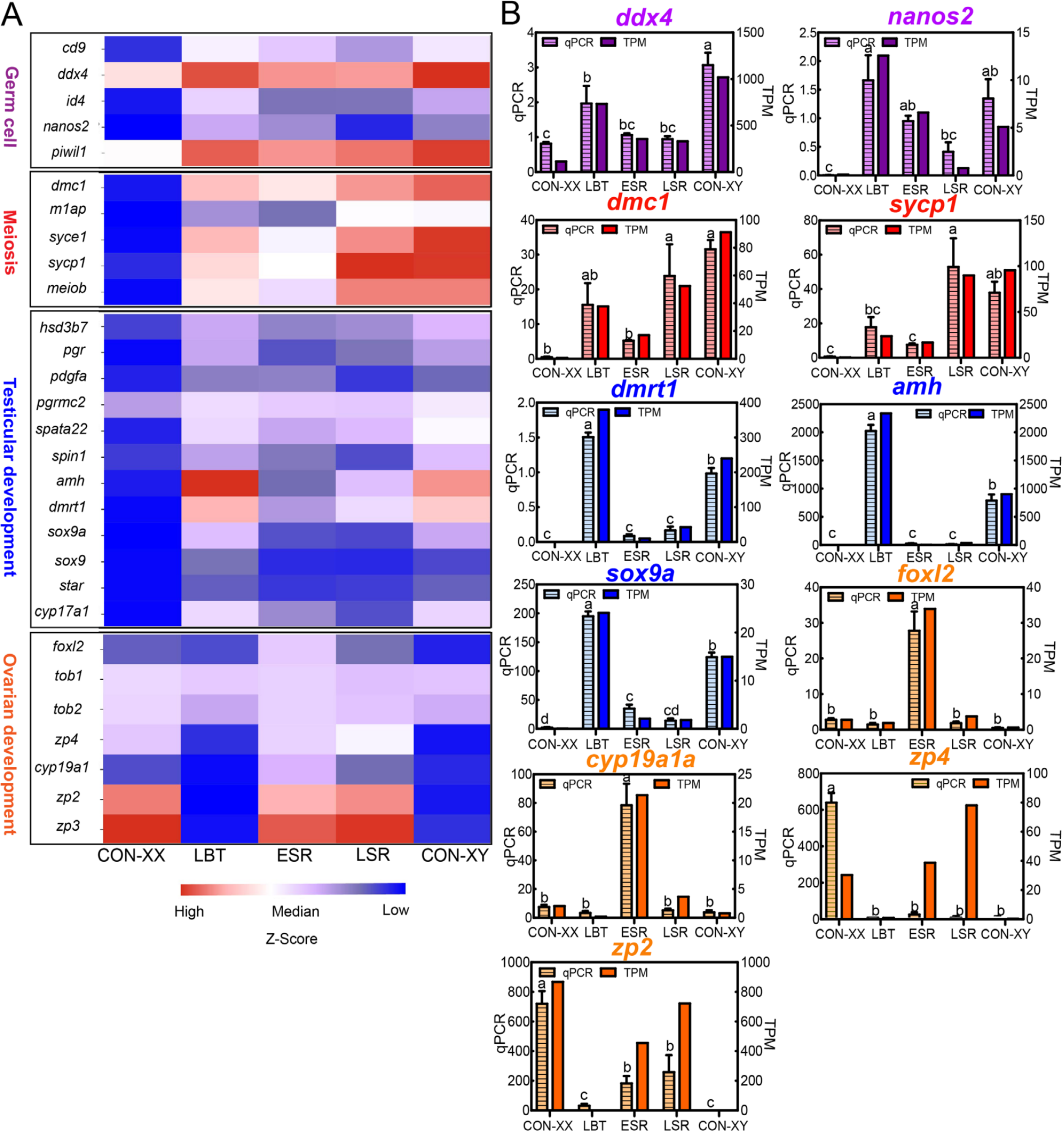
Temporal transcriptome of LTZ‐induced sex reversal. A) Heatmap showing expression of key genes related to germ cells, meiosis, testicular or ovarian development in control female (CON‐XX), late busulfan‐treated (LBT, 40 DBT), early sex reversal (ESR, 60 and 90 DAT), late sex reversal (LSR, 120 DAT), and control male (CON‐XY) gonads. B) RT‐qPCR validation and RNA‐seq expression values (TPM) for selected genes in CON‐XX, LBT, ESR, LSR, and CON‐XY gonads. Data are presented as mean ± SEM. Different letters indicate significant differences (*p*< 0.05), n = 3 each from three independent samples.

To delineate the transcriptional dynamics of gonadal sex reversal, we analyzed the top 500 transcripts in the ESR and LSR groups, which yielded highly concordant GO terms (Figure S5A,B). DEG analysis during sex reversal identified extensive transcriptional shifts. In the ESR group, 1437 genes were upregulated, and 518 were downregulated relative to LBT, while 2679 genes were upregulated and 362 were downregulated relative to CON‐XX. As sex reversal progressed, 568 genes were upregulated, and 59 were downregulated in the LSR (relative to ESR). Comparing CON‐XY, 3058 genes were upregulated, and 2890 were downregulated in the LSR (Figure S5C). Transcripts in the ESR group (relative to CON‐XX) were enriched in male‐specific reproductive processes and regulation of immune system processes (Figure S5D). Transcripts patterns indicated that germline stem cell markers (*cd9*, *ddx4*, *id4*, *nanos2* and *piwil4*) were among the earliest genes altered following LTZ treatment in the ESR (Figure 4A,B, Figure S5E). Interestingly, when compared to the LBT group, transcripts in the ESR group were enriched in GO terms associated with female‐specific transcripts with sperm‐egg recognition and binding of sperm to zona pellucida (Figure S6A). In line with this, we also detected a partial reactivation of ovarian pathways, evidenced by suppressed testicular markers (*amh* and *dmrt1*) and upregulated ovarian genes (*zp2* and *zp3*) (Figure 4A,B), indicating germline stem cell transcriptional plasticity during reproductive phases. In LSR, DEGs (relative to ESR) were enriched for spermatogenesis‐related terms, including microtubule‐based movement, cilium and motile cilium (Figure S6B). Furthermore, meiotic regulatory genes (*dmc1*, *m1ap*, *syce1*, *sycp1* and *meiob*) exhibited robust expression in the LSR group (Figure 4A,B). This suggested that LTZ‐mediated bimodal differentiation in BT‐treated ovaries, while concurrently promoting germline stem cell renewal and masculinization.

### 2.4 Somatic Sex Reprogramming from Ovaries to Testes

To investigate gonadal somatic cell reprogramming, we analyzed spatial patterns of key lineage‐specific markers including *foxl2* (granulosa cells) [5, 23], *cyp19a1a* (theca cells) [24], *sox9a* (Sertoli cells) [5, 15], and *hsd3b7* (Leydig cells) [15, 23] in BT‐ and LTZ‐treated gonads using immunofluorescence and in situ hybridization.

**FIGURE 5:**
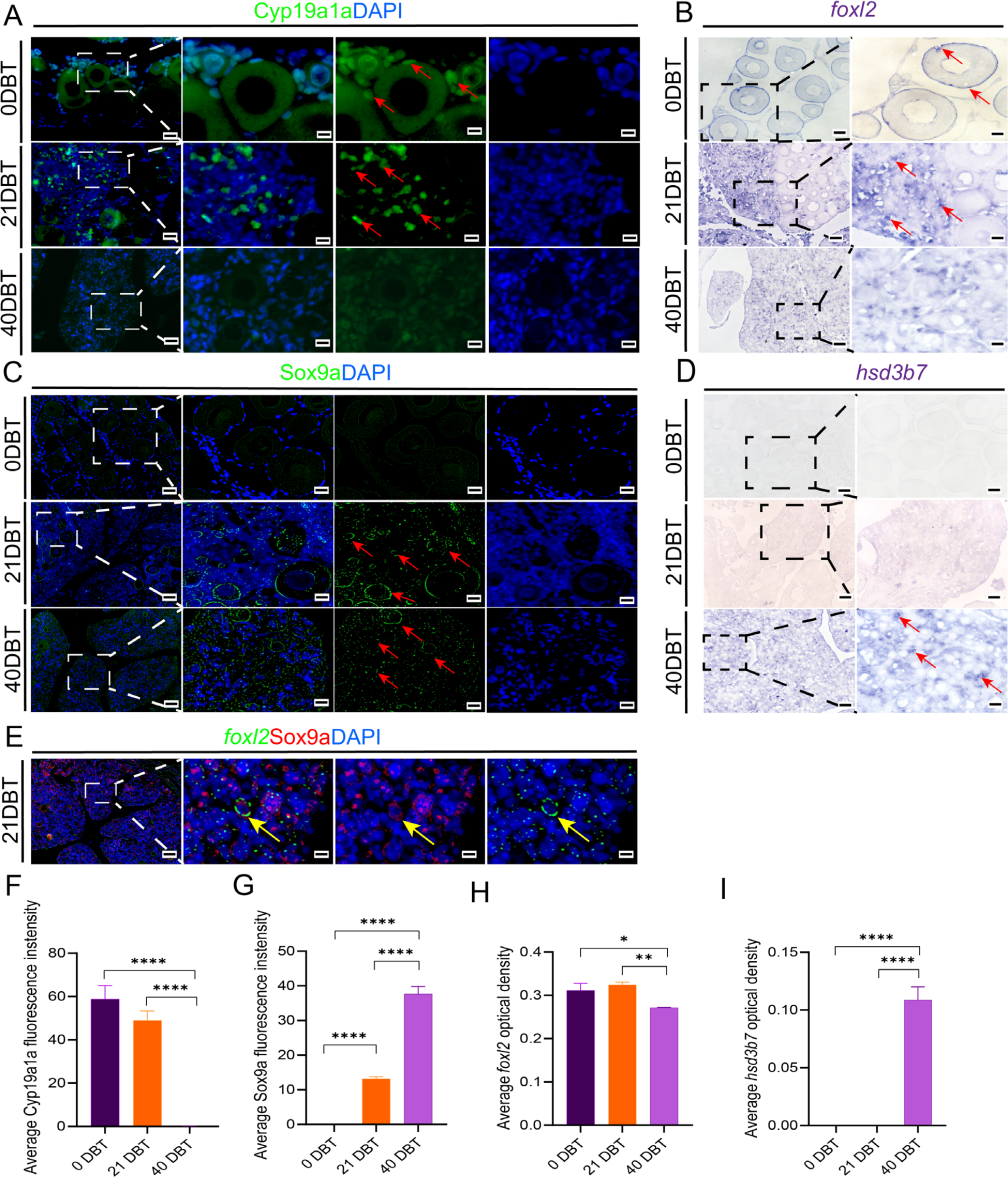
Oocyte ablation suppresses both Cyp19a1a and *foxl2* but promotes Sox9a and *hsd3b7* expression. A) Anti‐Cyp19a1a immunostaining showing theca cells (green; red arrow) in the gonads after busulfan treatment. Scale bars: 40 and 5 µm (high‐magnification insets). B) in situ hybridization of *foxl2* showing granulosa cells (blue; red arrow) in the gonads after busulfan treatment. Scale bars: 40 and 5 µm (high‐magnification insets). C) Anti‐Sox9a immunostaining showing Sertoli cells (green; red arrow) in the gonads after busulfan treatment. Scale bars: 40 and 5 µm (high‐magnification insets). D) in situ hybridization of *hsd3b7* showing granulosa cells (blue, red arrow) in the gonads after busulfan treatment. Scale bar. 40 and 5 µm (high‐magnification insets). E) Combination of fluorescent in situ hybridization of *foxl2* with immunofluorescence of Sox9a. Scale bars: 40 and 5 µm (high‐magnification insets). F) Quantification of average Cyp19a1a fluorescence intensity within the region of interest in gonadal tissues at 0, 21 and 40 DBT. *P*<0.0001(^****^), n = 3 each from three independent samples. G) Quantification of average Sox9a fluorescence intensity within the region of interest in gonadal tissues at 0, 21 and 40 DBT. *P*<0.0001(^****^), n = 3 each from three independent samples. H) Quantification of average *foxl2* optical density within the region of interest in gonadal tissues at 0, 21 and 40 DBT. *P*<0.05 (^*^), *P*<0.01(^**^), n = 3 each from three independent samples. I) Quantification of average *hsd3b7* optical density within the region of interest in gonadal tissues at 0, 21 and 40 DBT. *P*<0.0001(^****^), n = 3 each from three independent samples.

At 21DBT, BT‐treated ovaries exhibited abundant Cyp19a1a‐positive theca cells and *foxl2*‐positive granulosa cells (Figure 5A,B,F,H), resembling control ovaries. By 40 DBT, however, *foxl2*‐positive signals were significantly diminished (Figure 5B,H), while Cyp19a1a‐positive signals were entirely absent (Figure 5A,F), indicating an obvious loss of ovarian somatic identity. Conversely, SOX9A‐positive Sertoli cells and *hsd3b7*‐positive Leydig cells were observed in BT‐treated gonads by 21 DBT (Figure 5C,D,G,I), and were markedly upregulated at 40 DBT (Figure 5C,D,G,I), demonstrating somatic reprogramming toward a testicular phenotype. Notably, co‐spatial expression of *foxl2* and SOX9A in 21 DBT ovaries revealed transitional cell populations (Figure 5E), suggesting retained plasticity of terminally differentiated ovarian somatic cells.

**FIGURE 6:**
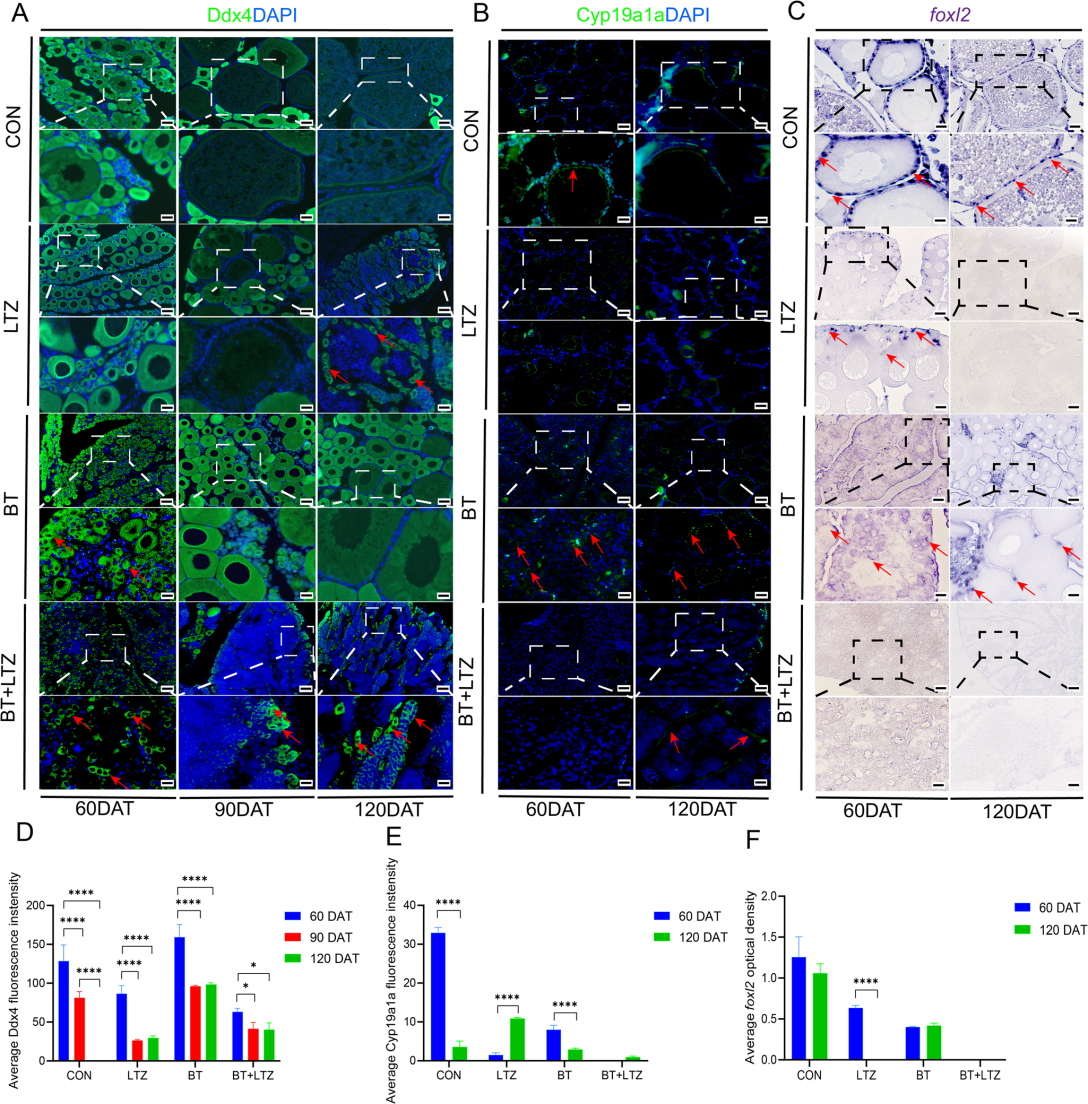
LTZ treatment suppresses Cyp19a1a and *foxl2* expression. A) Anti‐Ddx4 immunostaining showing oocytes and germline stem cells (green; red arrow) in gonads from the four experimental groups at 60, 90 and 120 days after treatment (DAT). Scale bars: 25 and10 µm (high‐magnification insets). B) Anti‐Cyp19a1a immunostaining showing theca cells (green; red arrow) in gonads from the four experimental groups at 60 and 120 DAT. Scale bars: 25 and 10 µm (high‐magnification insets). C) in situ hybridization of *foxl2* showing granulosa cells (blue; red arrow) in gonads from the four experimental groups at 60 and 120 DAT. Scale bars: 25 and 10 µm (high‐magnification insets). D) Quantification of average Ddx4 fluorescence intensity within the region of interest in the gonads of four experimental groups at 60, 90 and 120 DAT. *P*<0.05(^*^) and *P*<0.0001(^****^), n = 3 each from three independent samples. E) Quantification of average Cyp19a1a fluorescence intensity within the region of interest in the gonads of four experimental groups at 60 and 120 DAT. *P*<0.0001(^****^), n = 3 each from three independent samples. F) Quantification of average *foxl2* optical density within the region of interest in the gonads of four experimental groups at 60 and 120 DAT. *P*<0.0001(^****^), n = 3 each from three independent samples.

We next analyzed the spatial pattern of Ddx4 during LTZ‐induced sex reversal. At 60 DAT, BT+LTZ‐treated gonads exhibited abundant Ddx4‐positive spermatogonia (Figure 6A,D), with signals progressively restricted to the edges of gonads by 90 and 120 DAT (Figure 6A,D). In control ovaries, Ddx4‐positive signals were confined to primary growth oocytes (Figure 6A,D), whereas LTZ‐treated gonads retained Ddx4 signals at 60 and 90 DAT but showed a complete shift to spermatogonia within testicular lobules by 120 DAT (Figure 6A,D). Then, we assessed somatic cell transformation. In BT‐treated gonads, both Cyp19a1a and *foxl2* persisted through 120 DAT (Figure 6B,C,E,F). In contrast, BT+LTZ gonads lacked *foxl2* expression and displayed weak Cyp19a1a signals exclusively in the Leydig cells surrounding testicular lobules (Figure 6B,C,E,F). Notably, LTZ‐treated gonads exhibited strong *foxl2* expression on ovigerous lamellae at 60 DAT (Figure 6C,F), which was completely absent by 120 DAT (Figure 6C,F).

**FIGURE 7:**
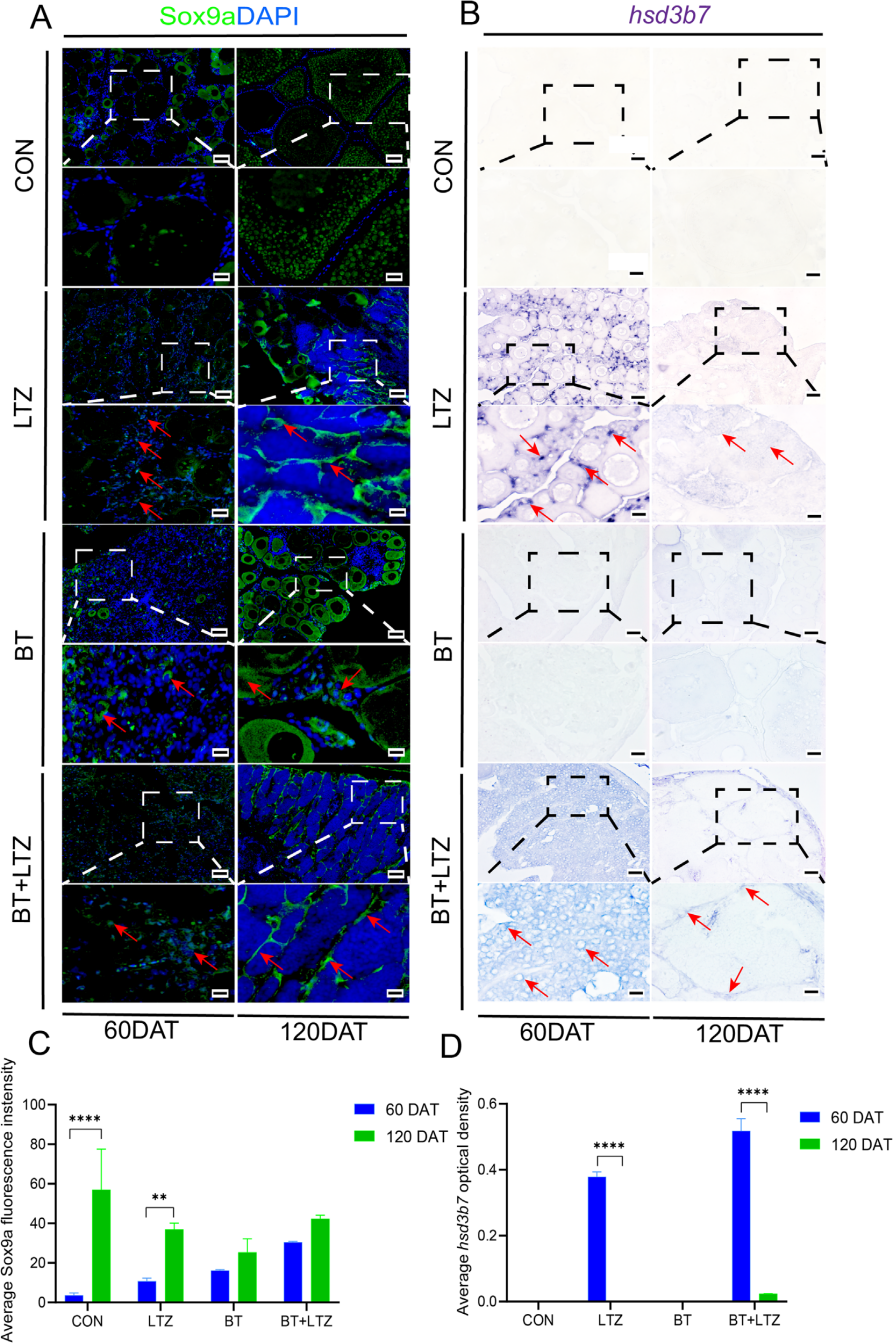
LTZ promotes Sox9a and *hsd3b7* expression. A) Anti‐Sox9a immunostaining showing Sertoli cells (green; red arrow) in gonads from the four experimental groups at 60 and 120 days after treatment (DAT). Scale bars: 25 and 10 µm (high‐magnification insets). B) in situ hybridization of *hsd3b7* showing granulosa cells (blue; red arrow) in gonads from the four experimental groups at 60 and 120 DAT. Scale bars: 25 and 10 µm (high‐magnification insets). C) Quantification of average Sox9a fluorescence intensity within the region of interest in the gonads of four experimental groups at 60 and 120 DAT. *P*<0.01(^**^) and *P*<0.0001(^****^), n = 3 each from three independent samples. D) Quantification of average *hsd3b7* optical density within the region of interest in the gonads of four experimental groups at 60 and 120 DAT. *P*<0.0001(^****^), n = 3 each from three independent samples.

Sox9a immunostaining revealed no detectable signal in control gonads, but was observed in BT‐ and BT+LTZ‐treated gonads at 60 DAT, with robust expression in Sertoli cells of BT+LTZ‐treated gonads by 120 DAT (Figure 7A,C). Complementary *hsd3b7* in situ hybridization showed no expression in BT‐treated gonads, whereas strong signals were detected in LTZ‐treated somatic cells at 60 DAT, and in BT+LTZ‐treated gonads at both 60 and 120 DAT (Figure 7B,D). These results demonstrate that oocyte ablation followed by LTZ treatment induces functional reprogramming of ovarian somatic cells into testicular somatic lineages (Sertoli and Leydig cells), driving complete ovarian‐to‐testicular structural and functional transition.

## 3 Discussion

**FIGURE 8:**
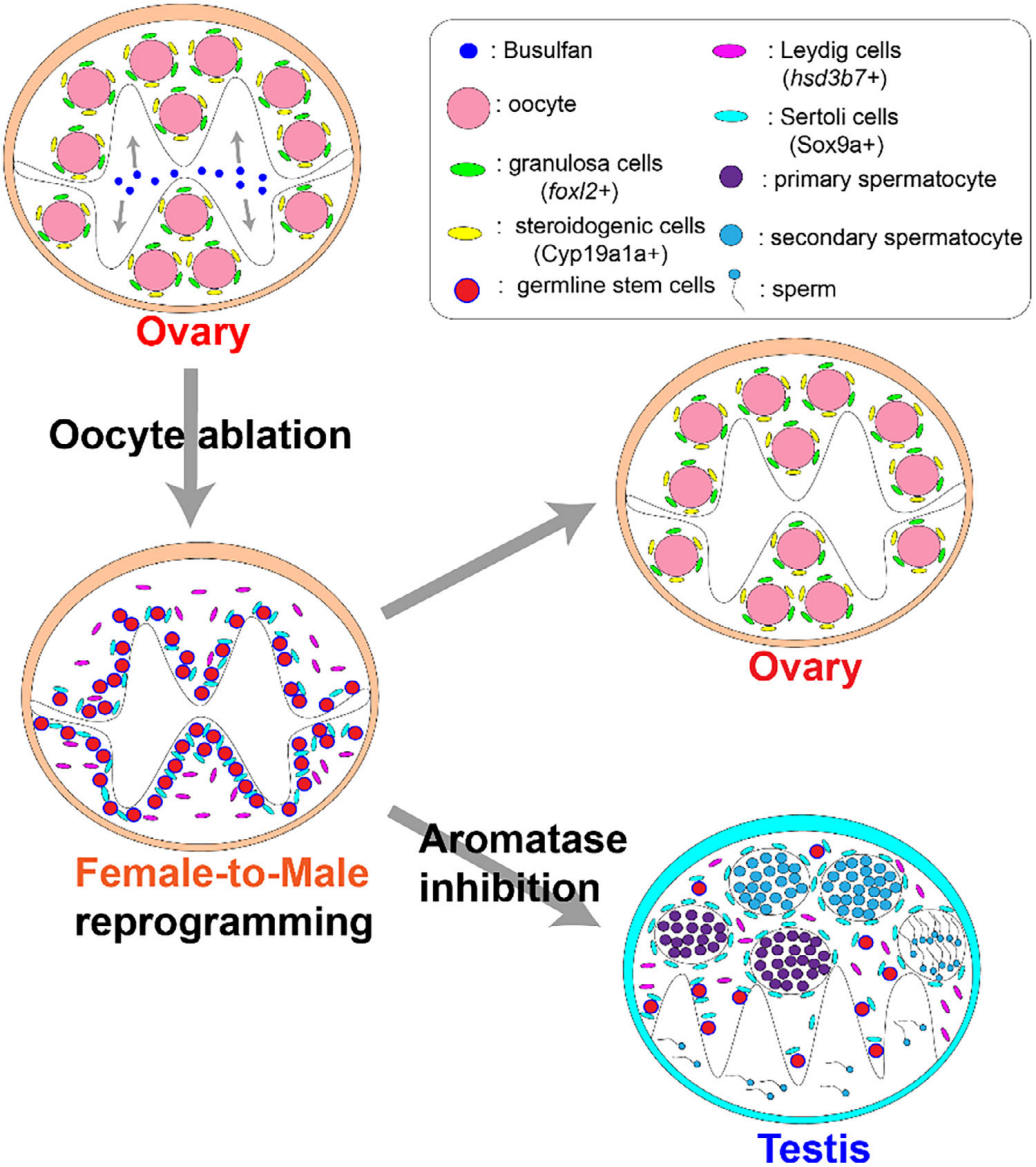
Model of secondary sex reversal in adult gonochoristic fish. In the adult ovary, oocytes maintain female fate. Intra‐ovarian busulfan treatment ablates oocytes, triggering a female‐to‐male somatic reprogramming where granulosa and theca cells transdifferentiate into Sertoli and Leydig cells. In the absence of further treatment, surviving germline stem cells (GSCs) regenerate oocytes, leading to ovarian recovery. However, if oocyte ablation is followed by aromatase inhibition (e.g., with letrozole), the GSCs are redirected to a spermatogenic fate, resulting in the formation of a functional testis capable of producing sperm.

Sex determination and gametogenesis depend on the interactions between germ cells and gonadal somatic cells. In mammals, the *Sry* gene, located on the Y chromosome, triggers the differentiation of the bipotential gonadal ridge into testes [1, 3]. In contrast, non‐mammalian vertebrates exhibit a broad variety of SD mechanisms, ranging from environmental to genetic systems, including multiple SD genes and even functional sex reversal [1, 6, 8]. Despite this diversity, the extent to which sex determination mechanisms retain plasticity or can be reprogrammed during adult stages remains poorly understood across vertebrates. In this study, we utilized *N. albiflora*, a marine teleost fish exhibiting a typical male heterogametic (XX/XY) system and multiple seasonal spawning events, to investigate the mechanism of secondary sex reversal from adult ovary to testis. We found that oocytes play crucial roles in maintaining ovarian fate. Furthermore, we identified ovarian germline stem cells in adult ovaries, which can undergo spermatogenesis and produce mature sperm under pharmacological induction. Importantly, we provide the first documented evidence of somatic transdifferentiation from an adult ovary to a testis in a gonochoristic teleost species (Figure 8). Collectively, our findings establish a novel model for exploring vertebrate sexual plasticity and offer a conceptual and technological foundation for advancing reproduction‐related manipulations in aquaculture.

### 3.1 Functional Sex Reversal via Oocyte Depletion in Adult Teleosts

In vertebrates, sex determination and gonadal differentiation typically occur during early stages of the lifespan. In mammals, the Y‐chromosome gene *SRY/Sry* directs the undifferentiated gonads to develop into testes, establishing the male phenotype [1, 17]. In fish, diverse sex‐determining genes have been identified, including *dmy*, *dmrtlby*, *amhy, gsdfy, amhr2*, *sdy*, *dmrt1* and *hsd17b1* [1, 2, 25, 26], yet germ cells usually regulate gonadal differentiation in most species [27, 28]. Germ cell depletion induces testis formation regardless of the genetic sex, such as in medaka (*O. latipes*) and zebrafish (*D. rerio*) [14, 27, 28]. In contrast, loach fish (*Misgurnus anguillicaudatus*) [29] and goldfish (*Carassius auratus*) [30], which lack germ cells, could develop as either phenotypic sex. These data suggest that the mechanism for sex determination of germ cells is varied among fish species. Recently, *foxl3* has been identified as a germ cell‐intrinsic factor controlling sperm‐egg fate decisions in fish [13, 31, 32]. However, key questions remain: Can a fully differentiated adult ovary transdifferentiate into a functional testis? What roles do the germ cells play in maintaining ovarian fate and enabling this plasticity?

Busulfan (1,4‐butanediol dimethanesulfonate) is a cell cycle‐nonspecific DNA alkylating agent originally developed for leukemia treatment and as a conditioning agent prior to bone marrow transplantation. While intravenous administration of busulfan has been shown to induce liver inflammation and damage through multiple pathways in mice [33], it is also known to selectively target proliferating germ cells with minimal off‐target impacts on gonadal somatic cells in both mammals and teleost fish [34, 36, 38, 39]. Consequently, it is widely utilized to eliminate germ cells and prepare recipients for germ cell transplantation in various animals, including mice [35], goats [36], pigs [37], and teleost fishes [40, 41]. The preservation of normal gonadal structure and successful production of donor‐derived gametes in these recipients further validate its efficacy and somatic safety [37, 40, 41]. Here, we developed an intra‐ovarian busulfan method to target‐deplete oocytes in *N. albiflora*. Histological and TUNEL analyses confirmed extensive oocyte apoptosis with preserved ovarian architecture (ovigerous lamellae and ovarian cavity). In protogynous hermaphroditism groupers [42] and juvenile hermaphroditism zebrafish (*D. rerio*) [43, 44], oocyte ablation induces ovarian‐to‐testicular sex reversal. Our data also demonstrate that oocyte depletion initiates gonadal masculinization in strictly gonochoristic species. This masculinization featured concurrent upregulation of testicular somatic gene expression (*hsd3b7*, *amh*, *dmrt1*, *sox9a* and *cyp17a1*) and suppression/loss of key ovarian transcript expression (*cyp19a1a*, *foxl2*, *zp4* and *tob2*). These results establish oocytes as commonly essential guardians of ovarian identity in fish. Their targeted removal induces masculinization, potentially supporting the hypothesis that hermaphroditism is evolutionarily derived from gonochorism. The oocyte itself regulates the development of ovarian follicles. Paracrine factors (e.g., growth differentiation factor 9, GDF9, bone morphogenetic protein, BMP15, and mechanistic target of rapamycin, MTOR) secreted by oocytes directly or indirectly govern the lineage, differentiation, and function of somatic cells [45, 46, 47]. Targeted depletion of these factors triggers the transdifferentiation of ovarian to testicular somatic cells. We hypothesize that oocyte‐derived factors play crucial roles in maintaining the function of somatic cells, but the precise genetic networks and signaling pathways driving this process warrant further investigation.

Steroid hormones critically regulate vertebrate gonadal differentiation. While estrogen/testosterone treatments during gonadal differentiation induce neofemale and neomale phenotypes in teleosts [22, 48], how estrogen maintains a female fate in gonochoristic adulthood remains unclear. In hermaphroditism species, ovary‐to‐testis sex change is correlated with decreased estrogen [42]. In gonochoristic species, such as medaka (*O. latipes*), Nile tilapia (*O. niloticus*), and zebrafish (*D. rerio*), prolonged aromatase inhibitor (AI, decreasing estrogen levels) treatment also triggers masculinization in mature females [10, 11, 49]. This suggests that the adult ovary retains a conserved secondary sex plasticity from gonochorism to hermaphroditism in fish species. Notably, our findings demonstrate that oocyte‐depleted females undergo rapid and complete ovary‐to‐testis reversal following a short‐term LTZ treatment. We define these sex‐reversed neo‐males as spermatogenically functional, exhibiting complete spermatogenesis and producing mature and motile spermatozoa. The assessment of in vivo fertilization capacity and offspring viability will be the focus of future investigations. We demonstrate complete sexual plasticity in adult gonochoristic teleosts and establish an efficient method to produce functional sperm from genetic females, enabling novel aquaculture biotechnologies.

### 3.2 Identification of Ovarian Germline Stem Cell‐Like (GSC‐like) Cells

Oocytes are essential for oogenesis and female reproductive function. In mammals, the primordial follicle reserve is established during early fetal development. At this stage, germ cells either enter meiosis, forming oocytes arrested in prophase I until puberty, or undergo apoptosis [50, 51]. Consequently, the number of oocytes available for ovulation is finite. For example, humans possess ∼1–2 million primordial follicles at birth but ovulate only ∼400 mature oocytes during their lifetime. In contrast, highly fecund fish maintain lifelong oocyte production [52, 53]. Their adult ovaries harbor mitotically active oogonia that sustain oocyte supply [52, 54]. These oogonia express conserved germline stem cell markers (Nanos2, Nanos3, and Ddx4), as shown in medaka (*O. latipes*) and zebrafish (*D. rerio*) [53, 55, 56]. While ovarian germline stem cells drive oogenesis, their role in adult ovarian‐to‐testis sex reversal was unknown. Previous studies using long‐term AI treatment to induce sex reversal in Nile tilapia (*O. niloticus*) and zebrafish (*D. rerio*) suggested the potential existence of GSCs in the adult ovary [9, 10]. However, the transdifferentiation of potential GSCs to spermatogenic cells and the process were unclear. In hermaphroditism species, the transdifferentiation of germ cells typically proceeds following oocyte depletion [8]. In this study, following oocyte depletion, we observed rapid and massive proliferation and masculinization of somatic cells, marked by the upregulation of male‐promoting network genes. Concurrently, the GSC‐like cells, which expressed the conserved germline marker Ddx4, were detected in the gonads. We considered these Ddx4‐positive cells to be a residual population, as the differentiated oocytes are incapable of dedifferentiate into GSC‐like cells. Meanwhile, our data suggest that the initial formation of masculinized somatic cells might provide a micro‐niche for the subsequent transdifferentiation of germ cells. Furthermore, the upregulated expression of GSCs‐related genes (*nanos2*, *piwil1*, *id4* and *ddx4*) might play important roles in maintaining the stem cell characteristics before the transdifferentiation of germ cells. In oocyte‐depleted gonads with BT, surviving ovarian GSC‐like cells developed into a large number of oocytes, demonstrating their role as the primary source of newly formed oogonia and oocytes in the adult ovary. In contrast, these GSC‐like cells exhibited active proliferation and further transdifferentiated into spermatogenic cells under the AI treatment, producing sperm. These data suggest that sustained masculinization of somatic cells with AI treatment induces the final sexual transdifferentiation of germ cells. This dynamic indicates that the fate of GSC‐like cells is regulated by the surrounding somatic cells. Taken together, we propose that ovary‐to‐testis sexual reversal and spermatogenesis present a model in which oocyte loss destabilizes ovarian identity and drives somatic masculinization, which in turn provides the niche permitting surviving GSC‐like cells to undergo spermatogenesis. Furthermore, we note that the present conclusion about these fate relationships is inferred from marker expression and temporal correlation, and the definitive lineage relationships between the surviving GSC‐like cells and the resulting spermatogenic cells remain to be established.

### 3.3 Somatic Cell Reprogramming in Ovary‐to‐testis Reversal

In vertebrates, gonadal somatic cells direct germ cell sexual fate commitment. During embryogenesis, somatic cells differentiate into testis‐specific (Sertoli and Leydig) or ovary‐specific (granulosa, theca) cell lineages [1, 57]. Sertoli and granulosa cells directly support germ cell development, while Leydig and theca cells produce sex steroids: testosterone in males and estrogen in females [58]. In mammals, loss of *dmrt1* in Sertoli cells activates *Foxl2*, reprogramming them into granulosa‐like cells [5]. Conversely, adult *foxl2* deletion converts ovarian somatic cells into testicular lineages [4]. However, somatic cell reprogramming during adult ovarian‐to‐testis reversal in gonochoristic fish remains unclear. In hermaphroditism species, some functional somatic cells of the ovary are thought to be reused as testicular somatic cells during the gonadal sex change [58, 59]. In AI‐induced masculinization of gonochoristic species, the female‐related genes (*cyp19a1a* and *foxl2*) were suppressed, and the male‐related genes (*dmrt1* and *sf1*) were activated. In this study, we directly demonstrate that oocyte depletion induces significant somatic cell lineage conversion, characterized by the loss of *foxl2*‐positive granulosa cells and Cyp19a1a‐positive theca cells, alongside the emergence of Sox9a‐positive Sertoli cells and *hsd3b7*‐positive Leydig cells. Notably, transitional cells co‐expressing Sox9a and *foxl2* indicate retained plasticity post‐differentiation, consistent with medaka studies showing granulosa and Sertoli cells derived from common embryonic precursors [13, 18]^.^ Furthermore, gonadal somatic cells exhibit bidirectional differentiation capacity, adopting either ovarian identities (*foxl2*‐positive granulosa cells and Cyp19a1a‐positive theca cells) or testicular lineages (Sox9a‐positive Sertoli, *hsd3b7*‐positive Leydig cells) under LTZ treatment. In mice, the phenotype of ovarian somatic cells was regulated by estrogen [60]. In this study, short‐term LTZ administration following complete oocyte depletion drives rapid and complete somatic reprogramming into male lineages. We hypothesize that gonadal somatic cells retain adult stem‐like properties enabling proliferation and sexual fate switching in the adult ovary; however, the specific genes and signal pathways regulating this transformation need further elucidation.

In protogynous fishes, previous studies showed that the induced testes will reverse to female after AI treatment was withdrawn. Furthermore, the gene expression of these transiently masculinized gonads exhibited no significant differences from those of control ovaries [11, 61]. Similarly, during the AI‐induced masculinization of gonochoristic species, the global gene expression profiles in the gonads also remained closer to the control female [10]. In contrast, research on the orange‐spotted grouper (*Epinephelus coioides*) has shown that the induced male sex can be stably maintained post‐treatment withdrawal if the gonads contain only male germ cells [62]. These findings suggest that establishing a globally male‐biased gene expression profile is critical for ensuring a stable testicular fate during adult ovary‐to‐testis sex reversal. In our study, the global transcriptomic profiles of sex‐reversed testes were found to be similar to those of control males. Furthermore, the successful production of functional sperm strongly indicates that the induced testicular phenotype is both functionally complete and stable. Future studies will focus on evaluating the fertilization competence of these induced sperm and the subsequent developmental viability of the offspring.

In summary, our findings demonstrate sexual plasticity in the adult ovary of a gonochoristic teleost species. The elucidation of molecular and cellular mechanisms underlying ovary‐to‐testis reversal establishes a new insight into sexual fate maintenance in vertebrates, and deepens our understanding of the general process of sex determination. Importantly, we have developed an efficient and reliable methodology for controlling sex manipulation in teleosts, with significant implications for aquaculture biotechnology. Furthermore, the identification of bipotent ovarian germline stem cells not only advances our understanding of germ cell biology but also establishes a valuable experimental system for investigating stem cell plasticity and their potential applications in reproductive technologies.

## 4 Methods

### 4.1 Animals

All animal experiments were approved by the Institutional Animal Care and Use Committee of Zhejiang Marine Fisheries Research Institute (approval no. [2023]21) and conducted in accordance with institutional animal ethics guidelines. Five hundred 8‐month‐old female yellow drum (body weight ∼150 g, body length ∼12 cm) were obtained from the research station of Zhejiang Marine Fisheries Research Institute (Xixuan Island, China) and used for the preparation.

### 4.2 Experimental Design

#### 4.2.1 Busulfan Treatments

Two hundred female yellow drums were anesthetized using tricaine methanesulfonate (MS222, MCE, cat. no. CS‐W012493) and immobilized on a wet sponge plate. Injection was performed using a micromanipulator (MP‐2R, Narishige, Japan) and microinjector (IM‐9B; Narishige) under a stereomicroscope (SZX10, Olympus, Tokyo, Japan). Blue ink was used to locate the urogenital papilla for the injection, and gonads were excised to confirm successful injection. Based on previous results, a dose of 40 mg/kg Busulfan (MCE, cat. no. HY‐B0245), dissolved in DMSO (Sigma–Aldrich, cat. no. D2438‐50ML), was selected. The busulfan solution was injected into the ovaries via the ovarian duct, which opens into the urogenital papilla, using a glass micropipette (outside diameter 70 µm). Approximately 100 µl of solution was injected into each fish. Injections were administered four times on days 0, 7, 14, and 21. After injection, fishes were reared under controlled conditions. During the experimental period, 60 fish were randomly sampled from 0 days after the first busulfan treatment (DAT), with 10 individuals at each sampling time (0, 7, 14, 21, 28, and 40 DAT) alongside untreated fish at 40 DAT.

#### 4.2.2 Letrozole Treatments

To examine the effects of busulfan treatment (BT) and letrozole (LTZ) on sexual maintenance, four experimental groups were established: (1) normal fish fed a normal diet as controls (CON, 3 tanks, n = 30 fish per tank); (2) genotypic females fed an LTZ diet (LTZ, 3 tanks, n = 30 fish per tank); (3) BT‐treated genotypic females fed a normal diet (BT, 3 tanks, n = 30 fish per tank); and (4) BT‐treated genotypic females fed an LTZ diet (BT+LTZ, 3 tanks, n = 30 fish per tank) (Figure 1A). From 40 days post‐BT treatment (DBT), fish were fed commercial feed with 100 mg/kg LTZ (Novon, China). LTZ was dissolved in 95% ethanol and applied to commercial feed pellets, which were then dried in a fume hood to evaporate the ethanol. Fish were fed four times per day to satiation. During the experimental period, 50 fish per group were randomly sampled from 0 DBT, with 10 individuals at each sampling time (0, 30, 60, 90 and 120 DAT). Before sampling, all fish were anesthetized with MS‐222, then the gonadal tissues were dissected quickly.

### 4.3 Histological Analyses

Gonadal tissues were fixed in Bouin's fixative solution for 24 h, dehydrated in a graded ethanol series, embedded in paraffin, and cut into 3–5 µm‐thick sections. Sections were stained with hematoxylin and eosin (HE) and examined and photographed using an Axio Imager A2 microscope equipped with an Axiocam 506 digital camera (Zeiss, Oberkochen, Germany). All 10 individuals from each sampling time were used for histological analyses.

### 4.4 Immunofluorescence Analyses

Gonadal tissues were fixed in 4% paraformaldehyde (PFA) overnight at 4°C, dehydrated in a graded ethanol series, and embedded in paraffin wax. Paraffin sections (5 µm) were deparaffinized and rehydrated in a graded ethanol series, and immersed in 1× universal powerful 125 antigen retrieval solution (Biosharp, China) for 30 min at a sub‐boiling temperature (95°C) for antigen retrieval. Sections were blocked for 2.5 h in blocking solution (10% goat serum in 1 × PBS) at room temperature, incubated overnight at 4°C with primary antibodies, washed five times with 1 × PBS (10 min each), incubated with secondary antibodies for 1 h at 37°C, and washed again with 1 × PBS. Primary antibodies included anti‐Ddx4 (1:1000, Abcam, cat. No. ab209710), anti‐PNCA (1:1000, Abcam, cat. No. Ab29), anti‐SOX9a (1:1000, Abcam, cat. No. AB209820) and anti‐Cyp19a1a (1:200). Primary antibody was detected using goat Anti‐Rabbit IgG H&L (Alexa Fluor 488) secondary antibodies (Abcam, cat. No. ab150077). Nuclei were counterstained with DAPI (10 µg/mL, Biosharp, cat. No. BL105A) for 5 min at room temperature, followed by three 5‐min washes with 1 × PBS. Fluorescence signals were observed under a BX‐51 N‐34FL fluorescence microscope (Olympus, Japan). Three independent individuals were used for each immunofluorescence analysis.

### 4.5 RNA‐seq

For RNA analysis, BT‐treated and LTZ‐induced sex‐reversal gonads were selected based on morphology. At 21 and 28 DBT, and 40 DBT, busulfan‐treated gonads were classified as early busulfan treatment (EBT) and late busulfan treatment (LBT) groups, respectively. At 60, 90 DAT, and 120 DAT, LTZ‐induced sex‐reversal BT‐treated gonads were identified as early sex reversal (ESR) and late sex reversal (LSR) groups, respectively. Total RNA was extracted using TRIzol reagent (Invitrogen, USA). RNA degradation was monitored in a 1% agarose gel, and RNA purity and integrity were measured using a Nanodrop2000 spectrophotometer (Thermo Scientific, USA). Library construction and sequencing were performed by the Experimental Department of Shanghai Majorbio Bio‐pharm Biotechnology Co. Ltd. (Shanghai, China). A total of 21 transcriptome profiles were constructed using the Illumina TruSeqTM RNA sample preparation Kit and Illumina Novaseq 6000 sequencing platform. Library preparation, sequence quality assessment, assembly, annotation, and differential expression gene (DEG) analyses were performed as described previously with minor modifications. Gene expression changes with |log2FC| >1 and adjusted *p*‐value ≤ 0.05 were considered significant. Transcripts per million (TPM) values were used for normalized counts in hierarchical clustering and temporal gene expression plots. Gene abundances were quantified using RNA‐Seq by Expectation Maximization (RSEM). All sequencing data are available through the NCBI Sequence Read Archive under the accession number PRJNA1321963.

### 4.6 RNA Extraction and qRT‐PCR

Total RNA from control and busulfan‐treated gonads was extracted using TRIzol reagent (Invitrogen, USA). RNA concentration was determined using the Qubit RNA Assay Kit with a Qubit 2.0 Fluorometer (Life Technologies). cDNA was synthesized using the PrimeScript RT reagent kit with a gDNA eraser (Takara, Japan) following the manufacturer's instructions. Expression levels of the germ cell markers (*ddx4* and *nanos2*), meiosis‐related genes (*dmc1* and *sycp1*), testicular development‐related genes (*amh* and *sox9a*), ovarian development‐related genes (*foxl2*, *cyp19a1a*, *zp4* and *zp2*), mitosis‐related genes (*pcna* and *ccnb1*) and apoptosis‐related genes (*p53*) were analyzed. Gene‐specific primers were designed using Primer Premier 5.0 and are listed in Table S1. *β‐actin* was chosen as a reference gene. qPCR amplifications were performed as previously described. The RT‐qPCR amplifications were performed using 2× SYBY Premix Ex TapTM II (Takara Bio) as described previously [63]. Three independent individuals were used for each qRT‐PCR. Each qRT‐PCR was performed in triplicate. The relative gene expression levels were analyzed using the 2^−ΔΔCT^ method.

### 4.7 In Situ Hybridization

Samples were fixed in 4% PFA and embedded in paraffin wax. Deparaffinized sections were processed for RNA in situ hybridization using the *foxl2* (XM_010731397.3) and *hsd3b7* (XM_010744954.3) probes as described previously [64]. For combined fluorescent in situ hybridization and immunofluorescence, deparaffinized sections were first hybridized with the *foxl2* probe as above and then processed for immunofluorescence with anti‐ SOX9a. Three independent individuals were used for each in situ hybridization analysis.

### 4.8 TUNEL Analyses

Apoptotic responses in gonadal tissues were evaluated using terminal deoxynucleotidyl transferase (TdT)‐mediated dUTP‐digoxigenin nick‐end labeling (TUNEL). Reactions were performed on sections (5 µm) of gonadal tissues fixed in 4% PFA‐fixed overnight at 4 ℃. Apoptotic cells were detected using Colorimetric TUNEL Apoptosis Assay Kits (cat. no. C1091, Beyotime Institute of Biotechnology, Shanghai, China) according to the instructions by the manufacturer. Images were captured using an Axio Imager 2 light microscope (Zeiss) and an LSM710 microscope (Zeiss). Image processing and analysis were conducted using AxioObserver (Carl Zeiss Microscopy GmbH, Jena, Germany) and ZEN software. Three independent individuals were used for each TUNEL analysis.

### 4.9 Statistical Analyses

Quantitative data are presented as mean ± SEM or box‐and‐whisker plots. Quantification of TUNEL+ cells/ total viable cells, average PCNA, Ddx4, Cyp19a1a, and Sox9a fluorescence intensity within the region of interest, and average *hsd3b7* and *foxl2* optical density within the region of interest was performed using ImageJ software. Statistical analyses were performed using GraphPad Prism 9 software. Two‐tailed Student's *t*‐tests were used for comparisons between two groups. For comparisons involving more than two groups, one‐way or two‐way ANOVA followed by Tukey's test was applied. Differences were considered statistically significant at *P*<0.05, with significant levels indicated as *P*<0.05 (^*^), *P*<0.01(^**^), *P*<0.001(^***^) and *P*<0.0001(^****^).

## Author Contributions

Y.Y., and D.X. generated hypotheses and designed the research. Y.Y., L.Y., R.C., and W.H. conducted experiments. Y.Y., and L.Y. analyzed the data. Y.Y. wrote the paper. Y.Y., D.X., W.Z., and G.W. revised the paper. All authors critically revised the report, commented on drafts of the manuscript, and approved the final report.

## Conflicts of Interest

The authors declare no conflicts of Interest.

## Supporting information




**Supporting File 1**: advs77027‐sup‐0001‐SuppMat.docx.

**Supporting File 2**: advs77027‐sup‐0002‐DataFile.xlsx.

## Data Availability

The data that support the findings of this study are available in the supplementary material of this article.
