# Supplementary material for: Functional Sex Reversal in Adult Gonochoristic Teleosts is Mediated by Ovarian Germline Stem Cell Plasticity and Somatic Cell Transdifferentiation
Source: Adv Sci (Weinh). 2026 Aug 15:e77027. Online ahead of print. doi: 10.1002/advs.77027 (PMC13477260; doi:10.1002/advs.77027)
Supplement: Supplementary file 1 [file ADVS-9999-e77027-s001.docx]

Supplementary figures

**Supplementary Figure 1. Busulfan-induced oocyte ablation, somatic cell proliferation and persistence of germline stem cells.** A) TUNEL staining showing apoptosis (brown; red arrow) in gonadal tissues following busulfan treatment. Scale bar. 100 μm and 15 μm (high-magnification insets). B) Anti-PCNA immunostaining showing proliferating somatic cells (green, red arrow). Scale bar. 50 μm and 15 μm (high-magnification insets). C) Anti-Ddx4 immunostaining showing oocytes and preserved germline stem cells (green, red arrow). Scale bar. 50 μm and 20 μm (high-magnification insets). D) Quantification of TUNEL+ cells/ total viable cells in gonadal tissues from 0 DBT to 40 DBT, n = 3 each from three independent samples. E) Quantification of average PCNA fluorescence intensity within the region of interest in gonadal tissues from 0 DBT to 40 DBT, n = 3 each from three independent samples. F) Quantification of average Ddx4 fluorescence intensity within the region of interest in gonadal tissues from 0 DBT to 40 DBT. *P* < 0.05 (*), *P* < 0.01 (**), *P* < 0.001(***) and *P* < 0.0001(****), n = 3 each from three independent samples..

**Supplementary Figure 2.** **Temporal appearance of apoptosis and proliferation during LTZ-induced sex reversal.** A) TUNEL staining for apoptotic signals (yellow, red arrow) in gonads from the four experimental groups at 60, 90 and 120 days after treatment (DAT). Scale bar: 100 μm and 15 μm (high-magnification insets). B) Anti-PCNA immunostaining showing proliferating cells in gonads from the four experimental groups at 60, 90 and 120 DAT. Scale bar: 100 μm and 15 μm (high-magnification insets). C) Quantification of TUNEL+ cells/ total viable cells in the gonads of four experimental groups at 60, 90 and 120 DAT, *P*<0.001(***) and *P*<0.0001(****), n = 3 each from three independent samples. D) Quantification of average PCNA fluorescence intensity within the region of interest in the gonads of four experimental groups at 60, 90 and 120 DAT. *P*<0.001(***) and *P*<0.0001(****), n = 3 each from three independent samples.

**Supplementary Figure 3.** **Transcriptomic analyses identified representative GO terms during busulfan treatment (BT).** A) PCA showing clustering and transcriptomic relationships among the six groups. B) The Euler diagram depicting the numbers of transcripts uniquely assigned to CON-XX, EBT, LBT and CON-XY. C) Representative GO terms enriched for these uniquely assigned transcripts.

 **Supplementary Figure 4. Differential expression gene (DEG) analyses identified representative transcripts during busulfan treatment (BT).** A) Bar chart showing DEGs in the following comparisons: LBT vs EBT, EBT vs CON-XX, and LBT vs CON-XY. B) Representative GO terms for the DEGs in LBT vs EBT. C, D) Representative GO terms for (C) upregulated and (D) downregulated DEGs in LBT vs EBT. E, F) Expression patterns for (E) downregulated and (F) downregulated DEGs in EBT, LBT, CON-XY and CON-XX groups. G) Heatmap displaying the expression of DEGs enriched in GO term “reproductive process” during BT.

 **Supplementary Figure 5. Transcriptomic analyses identified representative transcripts and GO terms during LTZ treatment.** A, B) Representative GO terms for the transcripts identified in (A) ESR and (B) LSR stages. C) DEGs in the following comparisons: ESR vs LBT, LSR vs ESR, ESR vs CON-XX, and LSR vs CON-XY. D) Representative GO terms for the DEGs identified in ESR vs CON-XX. E) Heatmap showing the expression patterns of DEGs enriched in GO term “reproductive process” during LTZ treatment.

**Supplementary Figure 6. Functional categorization of DEGs during LTZ treatment.** A, B) Representative GO terms for the DEGs identified in (A) ESR vs LBT and (B) LSR vs ESR. C, D) Representative GO terms for (C) upregulated and (D) downregulated DEGs in LSR vs CON-XY.

Supplementary Tables

Supplementary Table 1 The primers were used in this study

| Purpose | Gene name | Primers | Sequences (5’-3’) |
| --- | --- | --- | --- |
| qRT-PCR | *β-actin* | *β-actin*-F | GACCTGACAGACTACCTCATG |
|  |  | *β-actin*-R | AGTTGAAGGTGGTCTCGTGGA |
|  | *ddx4* | *ddx4*-F | GGAAGTGCCCTCGTGGTTAG |
|  |  | *ddx4*-R | TCCCAGTCATCGTCGTCAGC |
|  | *nanos2* | *nanos2*-F | GACACTGTACATAATTACCAAGAGG |
|  |  | *nanos2*-R | TAGACACTTGGGTTTTTACAAGTTT |
|  | *dmc1* | *dmc1*-F | TGCTGGACAACGTGCTTTAC |
|  |  | *dmc1*-R | TCATGGAATTTGGCTGCTAC |
|  | *sycp1* | *sycp1*-F | ACTCATAAATCACCAGGGAA |
|  |  | *sycp1*-R | TTCTTGTCACAGCCAATCAC |
|  | *amh* | *amh*-F | GTTGGCGGTGGATGTCTTCT |
|  |  | *amh*-R | ACAGGGTTAGGCTTGAGGGA |
|  | *dmrt1* | *dmrt1*-F | AAACCACGGCTATGTCTCAC |
|  |  | *dmrt1*-R | CGTCCTTCCATAGAGTACAAACAG |
|  | *sox9a* | *sox9a*-F | GCGTCAACGGCTCAAGTAAA |
|  |  | *sox9a*-R | CGCTCGGCTTCTTCCACAAA |
|  | *foxl2* | *foxl2*-F | AACCACCGTACTCTTATGTCGC |
|  |  | *foxl2*-R | CTGTTCTGCCAACCTTTCTTATTT |
|  | *zp4* | *zp4*-F | TTTGCCATCTTTCAGTTCCC |
|  |  | *zp4*-R | CCCTCTGCCTCCTTGTATGT |
|  | *zp2* | *zp2*-F | AGTGCCTTTCACGGACAGAG |
|  |  | *zp2*-R | AGACCGAGGGAGGAACTATG |
|  | *cyp19a1a* | *cyp19a1a*-F | TACAGGGCTCCTCGCCGTTAC |
|  |  | *cyp19a1a*-R | GCTGGGACAGGTTGTTGGTC |
|  | *p53* | *p53*-F | CAGTTGGGCTCTGAGATGACG |
|  |  | *p53*-R | TGAGGATGGGTCTGCGGTTC |
|  | *ccnb1* | *ccnb1*-F | GTTCACTTCCCACCTTCCAT |
|  |  | *ccnb1*-R | TCAGTCCCTCGTTTACCTTC |
|  | *pcna* | *pcna*-F | GAGTCAAGTTCTCCGCCACA |
|  |  | *pcna*-R | GACTGCCTCGTCCTCTTTGT |
| Genotype sex analysis | - | F | CACTTCTTGAGACCAGATTGTACAG |
|  | - | R | ACCTTGAAGTATTTCCGTGACAG |
